# Can Mindfulness Address Maladaptive Eating Behaviors? Why Traditional Diet Plans Fail and How New Mechanistic Insights May Lead to Novel Interventions

**DOI:** 10.3389/fpsyg.2018.01418

**Published:** 2018-09-10

**Authors:** Judson A. Brewer, Andrea Ruf, Ariel L. Beccia, Gloria I. Essien, Leonard M. Finn, Remko van Lutterveld, Ashley E. Mason

**Affiliations:** ^1^Center for Mindfulness in Medicine, Healthcare, and Society, Division of Mindfulness, University of Massachusetts Medical School, Worcester, MA, United States; ^2^Department of Quantitative Health Sciences, University of Massachusetts Medical School, Worcester, MA, United States; ^3^Contemplative Studies, Brown University, Providence, RI, United States; ^4^Needham Wellesley Family Medicine PC, Wellesley, MA, United States; ^5^Department of Family Medicine and Community Health, University of Massachusetts Medical School, Worcester, MA, United States; ^6^Department of Medicine, Osher Center for Integrative Medicine, University of California, San Francisco, San Francisco, CA, United States

**Keywords:** maladaptive eating behaviors, disordered eating, obesity, operant conditioning, reward, craving, mindfulness, mindful eating

## Abstract

Emotional and other maladaptive eating behaviors develop in response to a diversity of triggers, from psychological stress to the endless external cues in our modern food environment. While the standard approach to food- and weight-related concerns has been weight-loss through dietary restriction, these interventions have produced little long-term benefit, and may be counterproductive. A growing understanding of the behavioral and neurobiological mechanisms that underpin habit formation may explain why this approach has largely failed, and pave the way for a new generation of non-pharmacologic interventions. Here, we first review how modern food environments interact with human biology to promote reward-related eating through associative learning, i.e., operant conditioning. We also review how operant conditioning (positive and negative reinforcement) cultivates habit-based reward-related eating, and how current diet paradigms may not directly target such eating. Further, we describe how mindfulness training that targets reward-based learning may constitute an appropriate intervention to rewire the learning process around eating. We conclude with examples that illustrate how teaching patients to tap into and act on intrinsic (e.g., enjoying healthy eating, not overeating, and self-compassion) rather than extrinsic reward mechanisms (e.g., weighing oneself), is a promising new direction in improving individuals’ relationship with food.

## Introduction

Why do we eat when we feel stressed, anxious, or depressed? How does food craving play a role in the formation of eating habits? Can understandings the underlying mechanisms of these eating patterns explain why dieting fails, and lead to the development of novel and targeted interventions? In this article, we will address these questions.

## The Modern Food Environment Sets Us Up for Reward-Related Eating

Food- and weight-related issues are highly prevalent in the United States. Using 2016 data, the US Centers for Disease Control estimates the overall prevalence of obesity and overweight in US adults aged 18 years or older to be 29.6 and 35.2%, respectively (Centers for Disease Control and Prevention, 2016). While eating disorders, such as anorexia nervosa and bulimia nervosa are relatively rare (Smink et al., 2012), sub-threshold eating disorders are more common (Stice et al., 2009; Mangweth-Matzek et al., 2014) and disordered eating behaviors (e.g., binge eating) are prevalent among obese primary care patients (Chacko et al., 2015). Considering overweight/obesity and eating disorders as a spectrum, rather than as distinct and polarized conditions, has been hypothesized as a more effective approach to their treatment and prevention (Russell-Mayhew and Grace, 2016).

Empirical support for considering overweight/obesity and eating as a spectrum comes from recent research into eating psychology. Historically, two major maladaptive eating styles have been delineated: restrained eating (deliberate and persistent food restriction) (Herman and Mack, 1975) and disinhibited eating (an inability to inhibit eating once started) (Stunkard and Messick, 1985). Disinhibited eating is further divided into emotional and external eating, in which the former describes overeating in response to internal cues (i.e., emotions); while the latter describes overeating in response to external cues (i.e., seeing food that looks delicious) (van Strien et al., 1986). However, a growing body of evidence suggests that the distinctions between emotional and external eating are not as clear as previously assumed, and that they may represent a general concept of concerned and/or uncontrolled eating, characterized by low perceived self-control and high motivation to eat (Vainik et al., 2015; Bongers and Jansen, 2016). This is reinforced by recent findings indicating that emotional eaters tend to overeat in general (Bongers et al., 2016) it may be that such individuals tend to attribute overeating to negative affect (possibly due to mass media’s emphasis on emotional eating) (Adriaanse et al., 2016) when in reality, a plethora of cues can influence eating behavior, ranging from product placement at grocery stores, to frank messaging (e.g., “crafted for your craving”), to enticing commercial advertisements on billboards, television, and social media (Jansen et al., 2016).

Our modern food environment is replete with cues to both eat and not eat, as well as easy access to highly palatable foods (e.g., sugar-laden sweets). Such an environment plays a significant role in biasing control of eating behavior away from innate, internal processes (e.g., physiological hunger and satiety signals) to more external, artificial, or learned behavioral processes (e.g., seeing pictures of desirable foods). Continual exposure to such cues can alter our eating behavior in the short-term by triggering non-homeostatic eating (i.e., eating for reasons other than hunger) (Lowe and Butryn, 2007), or encouraging restriction despite physiological hunger (Polivy and Herman, 2017). While occasional episodes of over- or undereating should be considered part of “normal” eating behavior, over time, these cues may tap into our natural reward-based learning processes to cultivate habits of non-homeostatic eating and/or encourage recurrent binge-purge cycles in some populations (Burger et al., 2016). Perhaps unsurprisingly, many empirical studies have found correlations between habitual maladaptive eating behaviors and emotional duress, including depression, anxiety, and psychological stress (Appleton and McGowan, 2006; Ouwens et al., 2009; Miller-Matero et al., 2014; van Strien et al., 2016).

Here, we review what is currently known about the initiation and maintenance of maladaptive eating behaviors (henceforth referred to as reward-related eating) and how stress and emotions can amplify and/or stem from such behavior. We then review how traditional behavioral weight-loss dieting is insufficient in addressing reward-related eating mechanisms. Finally, we discuss how treatments that more directly target these mechanisms (with a focus on mindfulness training), may be promising strategies for reducing reward-related eating, and therefore its psychological and metabolic consequences.

## Mechanisms of Reward-Related Eating

**FIGURE 1:**
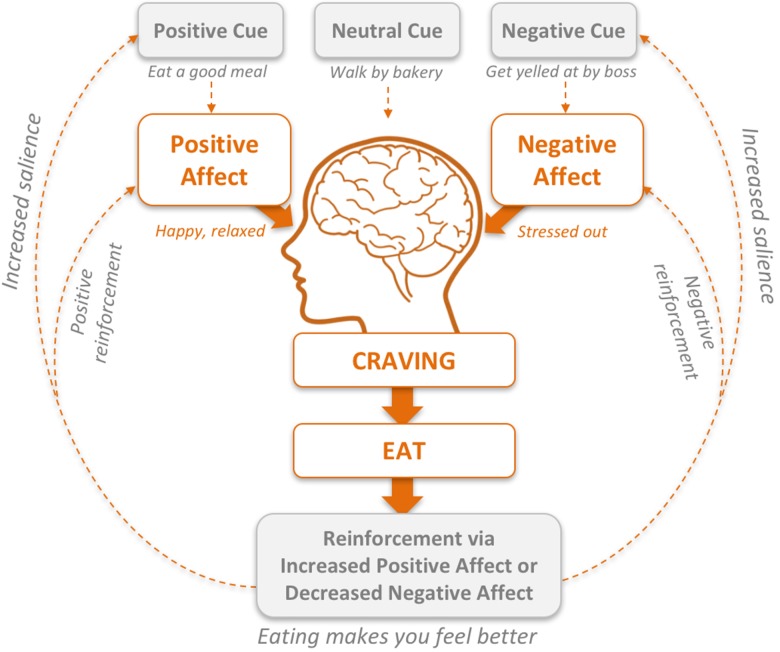
The habit loop. Development of habitual reward-based eating via positive and negative reinforcement.

From an evolutionary standpoint, it is adaptive to remember everything about good sources of food – when, where, and how to get them. To do this, we rely on one of the most well-characterized processes of learning: reinforcement or associative learning (i.e., operant conditioning). This includes both positive and negative reinforcement: the receipt of a reward or removal of a noxious stimulus, respectively, that increases the probability of repeating a behavior in the future (Epstein et al., 2007; Dayan and Niv, 2008; Singh et al., 2010). Behaviors learned via positive and negative reinforcement are reinforced by their consequences (rewards). Once our brains grasp the connection between a behavior and a reward, we create a powerful emotional memory that increases the probability of performing reward-yielding behavior in the future (Skinner, 1963). Put simply, if we eat a highly palatable food, we feel good, and lay down a memory that helps us remember under what circumstance we ate it, where we obtained it, what we liked about it, and so on. This memory reminds us to perform the same behavior the next time we are in a similar situation (positive reinforcement). Likewise, if we eat something that serves to reduce our sadness or anxiety, we may lay down a memory to eat certain foods to reduce particular affective states (negative reinforcement) (**Figure 1**). As such, in modern day, reward-related learning is still in play when food is not only plentiful (including a plethora of advertising to point us to its sources), but is also becoming more and more engineered to “hijack” the reinforcement learning system. Accordingly (and ironically), this evolutionarily conserved learning process has moved from helping us survive, to contributing to increased obesity-related morbidity and mortality.

Restrained eating may also be governed, in part, by operant conditioning. Women with eating disorders have been found to have an increased tendency to seek pleasurable experiences and avoid negative ones, which may underlie the binge-purge cycle (Smyth et al., 2007; Eneva et al., 2017). In regards to non-clinical populations, diet-related food cues (e.g., descriptions of “diet-friendly” food or pictures of thin bodies) tend to reduce food intake among already restrained eaters (Polivy and Herman, 2017), likely driven by the positive reinforcement of working toward or even reaching their body weight goals. Notably, restrained eating is associated with subsequent disinhibited, emotional, and/or binge eating (Polivy and Herman, 1985; Ricca et al., 2009; Péneau et al., 2013), which may be due to increasing the reinforcing value of food through repeated deprivation (Epstein et al., 2007). Such findings highlight the role of operant conditioning in influencing eating behavior across the spectrum of food-related issues.

There is evidence to suggest that repeatedly consuming highly processed foods (e.g., processed foods high in combinations of sugar and fat, salt and fat, or all three) can alter the brain’s reward circuitry. Such foods stimulates dopamine release along the same associative learning pathway as substances of abuse, and in some studies, this release surpasses that associated with cocaine use (Rada et al., 2005; Avena et al., 2006, 2008; Epstein et al., 2007; Lenoir et al., 2007; Stice et al., 2013). Although the concept of “food addiction” remains controversial, sugar and refined-carbohydrate consumption may lead to similar neuroadaptations as drugs of abuse, including craving and withdrawal (Ziauddeen et al., 2012). Repeatedly overconsuming sugar-laden food can condition individuals to expect pleasurable responses not only upon consuming a highly palatable food, but also when observing stimuli that one associates with the food (e.g., seeing a picture of ice cream) (Volkow et al., 2008). Such stimuli can activate learned associations that trigger non-homeostatic eating (Born et al., 2010; Dallman, 2010; Sinha and Jastreboff, 2013; Epel et al., 2014).

These positively and negatively reinforced learning pathways provide a useful explanatory model for why, how, and when people set up habits based on the rewarding experiences of eating and/or restricting, rather than true physical hunger (Kaplan and Kaplan, 1957; Schachter et al., 1968; Greeno and Wing, 1994). The more we engage in these habit loops by experiencing stress (trigger), eating palatable foods or restricting our eating (behavior), and receiving temporary relief (feeling better, being distracted from negative affect, moving toward a goal, avoiding feelings of guilt for having broken one’s dieting “rules” etc.), the further obscured our ability to recognize the difference between homeostatic and non-homeostatic hunger becomes.

Given the links between reward-based learning and maladaptive eating behaviors, it is surprising that to date, these positively and negatively reinforced habit loops have not been more explicitly incorporated into treatment paradigms for obesity and binge eating disorder. Specific aspects of the habit loop may provide direct and tangible targets for researchers and clinicians to develop and implement effective behavioral interventions that break the cycle of reward-related eating. As shown in **Figure 1**, craving is a central downstream component linking both positive and negative emotions to eating. Food cravings are most commonly defined as intense desires or longings to eat a specific food (Weingarten and Elston, 1991). Food cravings fit into a food reward framework as a psychological state of *wanting*, or appetitive motivation to seek out a particular food, which is distinct from liking, or the pleasure one derives from eating a particular food (Berridge, 2009). Psychological (rather than physical) deprivation is the more likely primary driver of food cravings (Polivy et al., 2005). Accordingly, theoretical frameworks, such as the Elaborated Intrusion (EI) Theory of Desires postulate that the conscious aspects of desire for a particular substance (i.e., a gracing) falls along a continuum of appetitive thought (May et al., 2012). Applied to food and eating, the EI Theory of Desire posits that cues to eat, be they cognitive, emotional, or physiological, can trigger seemingly spontaneous thoughts of images. These thoughts or images then motivate further elaboration and movement toward the desired food. Recent data map well onto this framework; for example studies show that food cravings predict non-homeostatic eating (Willner et al., 1998; Christensen and Pettijohn, 2001) and binge-eating (Joyner et al., 2015), and are associated with weight-preoccupation (Lafay et al., 2001).

## Willpower-Based Dieting to Reduce Reward-Related Eating: a Long-Term Weight-Loss Solution?

The standard clinical approach to weight-related medical issues is weight-loss, most commonly through dietary restriction. However, data have repeatedly demonstrated that traditional diet programs yield variable short-term results, and minimal differences in the long-term (Franz et al., 2007). For example, a recent systematic review and meta-analysis of 45 trials that examined the effects of long-term approaches for weight-loss maintenance found little evidence for the efficacy of lifestyle interventions (i.e., dieting) in maintaining weight-loss beyond 24 months (Dombrowski et al., 2014). As such, the outcomes of diet programs are notoriously poor; up to 60% of individuals regain all or more of the weight that was lost through dieting (Mann et al., 2007).

By definition, “effective” dieting requires vigilant self-regulation in order to make both short- and long-term decisions about food (Wing and Hill, 2001; DelParigi et al., 2007). However, the idea that one simply needs more willpower (where willpower is defined as the ability to resist shorter-term pleasures so as to achieve longer-term goals) to succeed on a diet may be suboptimal. Goal conflict theory suggests that the friction created by the desire to consume palatable foods and yet achieve long-term weight-loss goals, combined with incessant cues to eat in the modern food environment, sets the stage for self-regulation failures, leading to disinhibited reward-related eating (Hays and Roberts, 2008; Stroebe, 2008). As related to reward-based learning, willpower-based dieting strategies traditionally target the avoidance of cues, subversion of craving, and/or substitution strategies that treat “around” the core habit loop, rather than dismantling the loop itself. For example, one such method termed “attentional deployment” prescribes that individuals literally turn and focus their attention away from the craved food (Giuliani and Berkman, 2015). Although attentional deployment may effectively defer eating the food in that moment, it may not actually eliminate the craving itself, thereby allowing the craving to return when one’s willpower is depleted (Giuliani and Berkman, 2015). Importantly, many of these strategies depend on expending effort in the service of *reducing* craving-related eating, which to differing extents requires individuals’ willpower.

Factors that hamper willpower include cognitive exertion following demanding tasks (Vohs and Heatherton, 2000), attentional distraction, especially of the emotion-laden variety (Bechara, 2005; Heatherton and Wagner, 2011), and psychological stress (Arnsten, 2009). Furthermore, the presence of hunger, anger, loneliness, or/and tiredness (HALT) seems to promote a vulnerable state for self-regulatory failure (Vohs et al., 2005; Mead et al., 2009; Arnsten, 2015). Collectively, these findings suggest that maladaptive eating behaviors are not simply “food” problems, and thus interventions that treat them as such may exacerbate the issue. For instance, some interventions have sought to bolster self-regulatory resources by requiring new behaviors, such as daily self-weighing (e.g., Wing et al., 2006) so as to reduce decision-making. A major limitation of these interventions is that these attempts at automation often require too much effort to sustain (and, in many cases, even initiate) – especially when they can feel punitive in nature (ironically, which can induce negative affect). Other researchers have developed “small changes” or “behavioral nudge” interventions that focus on reducing triggers in the environment that tax willpower (Hill, 2009). Although these environmental strategies show promise (e.g., Eldridge et al., 2016), it is impossible to manipulate or otherwise control the environments everywhere one goes.

Perhaps most importantly, construing reward-related eating as a lack of willpower ignores the biology underlying restriction and cultural context in which such behaviors develop. Mechanistically, recent research suggests that weight-loss through dietary restriction is accompanied by hormonal and metabolic adaptations that promote weight regain through increased appetite (MacLean et al., 2011; Fothergill et al., 2016). In addition to these biological influences is a paradoxical combination of an obesogenic food environment situated within a culture that emphasizes thinness for both health and esthetic purposes. Such an environment has been found to produce a conflicting set of social norms surrounding food and weight among women (Whale et al., 2014), which may contribute to, and reinforce, maladaptive eating behaviors.

Thus, interventions predicated on external methods (e.g., changing our environment) or on cognitive methods (e.g., willpower) that do not directly target the habit loop (e.g., prescribing restrictive behaviors) have not resulted in reduced reward-related eating, and for some, may be counterproductive. As the mechanisms of reward-related eating are now becoming clearer, can these insights inform currently employed diet and behavior change interventions? Investigating intervention modalities that *directly target* key elements of the habit loop (e.g., craving), as compared to attempting to use cognitive strategies to change them or treat around them (e.g., substitution), may inform the development of more effective ways to sustainably reduce reward-related eating.

## Addressing Craving From the Inside Out: the Role of Mindfulness

We have previously found that with habitual behaviors, such as smoking, craving has been shown to be a critical link of the habit loop (Brewer et al., 2013b; Elwafi et al., 2013). Similar to how craving palatable food can lead to non-homeostatic eating, craving for cigarettes significantly predicts smoking (Brewer et al., 2011; DiFranza, 2016). Interestingly, interventions such as mindfulness training have historical roots in targeting and managing craving itself, rather than treating “around it” through the use of substitute or avoidance strategies as described above, suggesting a theoretical overlap between ancient and modern mechanisms (Bhikkhu, 2013; Brewer et al., 2013b). Mindfulness can be defined as the awareness that arises when paying attention in the present moment, on purpose and non-judgmentally (Kabat-Zinn, 2006). Another common definition of mindfulness used in research includes two components:

(1) Self-regulation of attention so that it is maintained on immediate experience, thereby allowing for increased recognition of mental events in the present moment, and (2) Adopting a particular orientation toward one’s experiences in the present moment, characterized by curiosity, openness, and acceptance (Bishop et al., 2004). In other words, “being mindful” means allowing experiences to unfold with curiosity rather than with attempts at control, which may enable healthier management of issues relating to affect-driven cravings (Brewer and Pbert, 2015).

We have found that mindfulness training directly targets reward-based habit loops (Brewer et al., 2013b). For example, smokers who underwent mindfulness training quit at five times the rate of smokers who received the American Lung Association’s Freedom from Smoking program, which is based in cognitive strategies (Brewer et al., 2011), likely due to a decoupling of the association between craving and smoking (Elwafi et al., 2013). In other words, individuals learned to pay attention to and “be with” their cravings instead of compulsively acting on them or painfully struggling with them (Brewer and Pbert, 2015; Brewer, 2018). Importantly, *this is fundamentally different* than other cognitive techniques targeting cravings. Instead of changing, suppressing, resisting, or avoiding cravings, mindfulness helps individuals accept and paradoxically move closer to the thoughts, emotions, and body sensations that make up cravings. This enables individuals to discover how cravings are driving them to act, and in doing so, learn to tap into the very same reward-based learning system to gain mastery over them. Herein, mindfulness may lead to reductions in cravings over time through extinction, rather than suppression (Tapper, 2018).

### Next-Generation Mindfulness Training for Reward-Related Eating

Mindfulness training has been shown to reduce maladaptive eating behaviors (e.g., emotional eating, external eating, binge eating, reactivity to food cravings, restrained eating, and mindless eating) across a majority of studies (Godsey, 2013; Katterman et al., 2014; O’Reilly et al., 2014; Godfrey et al., 2015). How might mindfulness training help individuals improve their relationship with eating? Might it target the habit loop in a similar manner to what has been shown with breaking habits, such as smoking? As craving may be a core mechanistic link in reward-based learning, there may be ways to specifically target mindfulness training to the actual mechanisms driving eating.

Below, we outline three broad steps that individuals take as they learn to be mindful of their eating habits (increasing awareness, evaluating outcomes, and making embodied choices), and provide real-world examples from participants in a newly developed digitally delivered mindful eating program that specifically employs these as a way to target reward-based eating (**Table 1**).

**Table 1:** Examples of experiences with each of the three steps of the mindful eating model, provided from program participants within the smartphone application platform.

1. Awareness	“I gained insight today relating to the correlation between my exercise routine and my eating patterns”.
	“I am really seeing how the habit loop has driven my life with food”.
	“I just realized how my internalized anger, resentment, and self-deprecation are expressed in my eating”.
	“It has been so helpful to gradually learn to return again and again and again without criticizing myself. I’m beginning to see how that same practice might help me with my eating”.
2. Evaluating outcomes	“I’m finding that I’m listening to my body, noticing how my feelings are sensations in my body. I’m also tasting my food, and learning what taste [sic] good and doesn’t. I can already feel the habit loops leading to eating being interrupted. I don’t fight with myself all day long, either winning the food battle or losing it”.
	“I got beyond thoughts of the rewards alone of my craving and reflected on the consequences. Once it hit me that satisfying my craving wouldn’t fulfill my needs, wouldn’t solve the problem and would in fact only make me feel worse, I began looking at it as less desirable an action”.
	“A shift is happening; I’m choosing more healthy foods. The sugary things are less attractive. Satiety is now coming into focus”.
	“Had a piece of chocolate and ate it mindfully, what a difference! Normally it is just eaten quickly and in reality not enjoyed. As it turned out, the one piece was sufficient, that’s normally not the case. Small win”.
3. Unforced, embodied choice	“I wasn’t going to make myself try to eat less but just showing up and being as present with the experience as I could be. That helped a lot and then I ordered my food and really tried to be there and see what I was eating and feeling and experiencing”.
	“I am feeling like I can tune into what my body needs more now my emotions around food are more settled. The protein powder with berries for breakfast was filling and set me up for the day. I tuned in to my body in the late afternoon and just wanted a banana and a few nuts – I felt like these carbs were ok and went with my intuition”.
	“It’s a birthday party. Food all over the place. Pizza, salads, butter, and caramel cupcakes. With the powerful artillery of mindfulness and RAIN, I managed to enjoy a little bit of pizza, satisfactory portions of healthy salads and half a cupcake, shared with my daughter. I felt in control for the first time, I was Superman!”

#### Step 1: Awareness – We Cannot Change What We Cannot See

We hypothesize that the first step in changing habitual eating behavior is becoming aware of such behaviors and their triggers. Maladaptive eating patterns are often learned and reinforced for years. For example, children may learn to pair food with emotional rewards (e.g., parental approval) (Farrow et al., 2015), and 63% of children aged 5–13 have reported eating in response to mood (Shapiro et al., 2007). Thus, reward-related eating can become ingrained early in life. As such, many individuals report that they do not notice that they are out of touch with their hunger and satiety signals until they are experiencing consequences, such as the physical effects of feeling overly full or extreme hunger. A clear recognition of elements within the habit loop (i.e., triggers and behaviors) can help people to begin working with them, rather than continuing to reinforce the habitual maladaptive behaviors. This recognition is one of the core principles of many mindful eating programs that are delivered in person (Rossy, 2016). **Table 1** presents participants’ self-reports about their experiences while gaining awareness, illustrating how this newfound awareness often helps people eat when they are physiologically hungry and not reinforce reward-related eating.

#### Step 2: Evaluating Outcomes – Clearly Seeing the True ‘Rewards’ of Our Habits

The second step in changing habitual eating is a clear recognition of the actual results (rewards) that one is receiving from the behavior. Specifically, these are the direct physical sensations and emotional effects of eating beyond satiety or when triggered in the absence of hunger. This step taps directly into and utilizes reward-based learning itself. Early theories underlying mindfulness training suggest that such clear and unbiased recognition is a critical step for lasting habit change (Brewer et al., 2013a). By evaluating results or outcomes, we mean an accurate assessment of *everything* that results from an episode of reward-related eating, rather than selectively paying attention to only certain aspects of the experience. For example, if one eats to numb themselves from painful feelings and only attends to the temporary relief, they may not remember accompanying physical feelings, such as being uncomfortably full and lethargic, or resultant emotional aspects of the experience, such as feelings of guilt.

Non-judgmental awareness of the entire experience provides an opportunity to “add up” all of the elements resulting in a more accurate calculation of the sum total of the reward. Outcome evaluation begins a process of disenchantment with habitual behaviors, as a thorough assessment of the rewards reveals that they are not as rewarding as once perceived. Importantly, this evaluation is not an intellectual interrogation (e.g., “I shouldn’t have eaten this because I will gain weight”), but rather an exploration of one’s immediate experience (e.g., “wow, I feel sick, [and guilty]”). Linking action to experiential outcome is critical for updating the neural reward-value of one’s behavior in the orbitofrontal cortex (Kringelbach, 2005), tapping into the very reward-based learning process that set up the unhealthy behavior in the first place, rather than relying on will-power or cognitive control regions of the brain (e.g., lateral prefrontal cortex), which are susceptible to failure in times of stress and hunger (Arnsten, 2015).

This same process can be employed when adopting new eating behaviors, allowing one to bring awareness to the experience so as to appreciate the physical and psychological effects of eating when truly hungry (while also enjoying the experience) and stopping when full. In pilot studies of brief mindfulness interventions, hints of carryover effects have even been seen in which individuals who eat a meal mindfully consume 45% fewer calories while snacking 2 h later (Seguias and Tapper, 2018), likely due to a heightened ability to sense internal cues relating to hunger and satiety. Disenchantment with prior maladaptive eating behaviors combined with the learning of mindful eating fosters the development of an embodied wisdom-based eating framework (described in detail below), rather than a cognitive, knowledge-based one. This learning process may be critical for long-term and sustainable behavior change, as it draws from one’s own experiences, unlike standard cognitive based weight-control strategies. Illustrative examples of participants’ experiences with this process are presented in **Table 1**.

#### Step 3: Unforced Freedom of Choice – Supporting Intuitive Self-care

The third step in changing habitual eating is developing the ability to make unforced, embodied choices about food. The framework and specific language for this step was derived from qualitative data from focus group discussions with participants of the mindful eating program, based on their direct experience (Beccia et al., in preparation). Based on our findings, step three was defined as unforced freedom of choice, emerging from embodied awareness, in the present moment. In other words, an awareness of the links between behavior and outcome cultivates a heightened ability to make “intuitive” choices that support self-care in a way that feels effortless, rather than forced. The intuitive sense emerges directly from the disenchantment learned in step two, such that one consciously or unconsciously compares the relative rewards from these previous actions to guide current behavior. Notably, there have been calls to implement interventions that support self-care and healthy lifestyles, particularly ones that are patient-centered, within primary care settings (Greaves and Campbell, 2007); this model of mindful eating represents such an intervention, as it helps individuals move away from the “shame and blame” thinking that comes with cognitively based dieting (“I should eat X,” I shouldn’t have eaten Y,” etc.), and into more self-compassionate ways of being.

This is critical, as many individuals spin out into cycles of shame and blame when stepping onto the scale or looking in the mirror, which ironically often triggers “eating-to-cope” habit loops. Self-compassion has been proposed to amplify the effectiveness of mindfulness, and preliminary evidence suggests that self-compassion promotes intuitive eating and other positive health behaviors (Mantzios and Wilson, 2014, 2015). Being compassionate toward oneself builds on the exploration of the results of self-judgment as part of step two (e.g., seeing that self-flagellation or guilt does not feel good), and importantly, can be deliberately fostered. For example, self-compassion is formally taught in our mindful eating program through loving-kindness practices directed toward oneself, and is specifically framed in the context of the habit loop as an alternate to emotional eating. In this way, individuals can contrast the differential results from compassion versus self-judgment. Over time, as the relative rewards of self-compassion become more evident and accessible, this type of self-care becomes more “intuitive,” driven by the updating of its reward value in the orbitofrontal cortex (as noted earlier).

Importantly, and in line with some of the earliest reports of mindfulness training (Kabat-Zinn, 1982), mindfulness may constitute a different form of self-regulation than the self-control that comes with cognitive or deliberate effort – one that is fostered by an “effortless awareness” (Friese et al., 2012; Garrison et al., 2013; van Lutterveld et al., 2017). While attempting to use cognitive control to resist, fight, or distract oneself from the experience of craving precludes changing a problematic habit loop (Vohs and Heatherton, 2000), an unforced, curiosity-based observation of its elements and their time-course may decrease the likelihood of falling back on previously learned behaviors (including self-judgment). We have found with mindful eating as well as smoking cessation programs that using in-the-moment exercises, such as **RAIN** (**R**ecognize the craving, **A**llow it to exist, **I**nvestigate what it feels like in the body, **N**ote the associated physical sensations from moment-to-moment) gives pragmatic tools for observing and even co-existing with cravings rather than using cognitively based suppression or avoidance techniques (Elwafi et al., 2013). This open investigation supports the close investigation of what physical sensations make up cravings, bringing one into her or his own experience, which is often experienced as pleasant (or less unpleasant) compared to being caught in the grip of a craving.

In sum, through this three-step progression, mindfulness training can directly target core aspects of reward-based learning, and even tap into this very process to update the reward-value of habitual eating behaviors. Such training improves one’s relationship with food by facilitating present moment awareness of one’s direct experience, and may result in lasting behavior change.

#### Digital Therapeutic Delivery of Mindfulness Training

Based on the reward-based learning model described above, we developed a mindful eating program that can be delivered via smartphone with online community support [described in detail in Mason et al. (2017)]. Using short daily trainings delivered via video, audio, and animations, as well as in-the-moment exercises, this program promotes training in mindfulness skills *within the actual environment* in which one develops and reinforces habitual eating patterns. This intervention first empowers individuals to understand how they form habitual eating patterns (i.e., the habit loop) and to clearly see what “rewards” they are receiving from their behavior. Similar to our app-based training for smoking cessation (Garrison et al., 2015), this mindful eating intervention teaches individuals mindfulness tools in a step-by-step manner to help them change their habitual responses to food cravings and realign eating with physical hunger and satiety cues.

Some of the original in-person mindful eating programs begin with an emphasis on mindfulness meditation practices as a way to foster the development of non-judgmental awareness of automatic patterns related to eating (e.g., Kristeller and Wolever, 2011). Although those programs and ours are theoretically and conceptually aligned, data from our early studies with smoking cessation suggested that short, informal, in-the-moment mindfulness practices (e.g., RAIN) yielded greater decoupling of craving and behavior than more formal meditation practices (e.g., sitting meditation) (Elwafi et al., 2013). Accordingly, we specifically developed the program to emphasize short, momentary mindfulness practices directly related to the habit loop in one’s everyday life that are subsequently supported by more formal meditation practices as awareness and mindfulness skills are developed.

These principles are based on the same tools we have shown to moderate the decoupling of craving and smoking behavior in previous clinical trials focused on craving-related habits (Elwafi et al., 2013), and are yielding early empirical evidence for decoupling craving and eating. For example, we administered our 28-day smartphone-delivered mindful eating program to 104 overweight or obese women, and found that the women experienced significant reductions in both craving-related eating (40% reduction, *p* < 0.001) and overeating behavior (e.g., 36% reduction in eating to cope with negative emotions, *p* < 0.001) (Mason et al., 2017).

## Conclusion

The prevalence and consequences of obesity are frequently highlighted; lesser discussed are maladaptive eating behaviors, such as restrained, emotional, and binge eating that can have serious physical and psychological effects. While the standard approach to food- and weight-related health and disease issues is dietary restriction to achieve weight-loss, we contend that such an approach is inadequate at best and counterproductive at worst. There is a growing body of evidence suggesting that it is possible to improve a range of health outcomes (including metabolic risk factors, heart disease, hypertension, depression etc.) independent of weight-loss, likely through enhancing behaviors relating to diet and activity (Bacon and Aphramor, 2011; Schaefer and Magnuson, 2014; Tylka et al., 2014; Van Dyke and Drinkwater, 2014). Given the well-established challenges in maintaining long-term weight-loss (Wing and Phelan, 2005; Dombrowski et al., 2014), as well as the social consequences of emphasizing weight, including prevalent weight-based discrimination (Spahlholz et al., 2016) and the normalization of body image discontent (Tantleff-Dunn et al., 2011), adopting strategies to improve eating behaviors that mitigate the issues inherent in dietary restriction should be a priority to healthcare providers.

In this article, we have provided the theoretical framework and early empirical evidence for an intervention that meets these criteria. Mindful awareness of habitual, maladaptive eating behaviors may help people to improve their relationships with food. When people have a clear window through which to view how habit loops are developed (e.g., eating when stressed) and maintained (e.g., reward-based learning), engaging in interventions that directly disrupt these loops (such as the mindful eating program we have described) can be an empowering process. That is, honing interventions to directly focus on core elements of the habit loop, rather than developing behavioral workarounds, may affect more lasting change.

Additionally, the recalibration of rewards that results from mindfulness training may provide a novel way to reframe the “diet” process. Focusing on intrinsic rewards, defined as those coming from our own experience of being mindfully engaged with a process (e.g., savoring food, noticing the rewards of healthy eating, and stopping when full), may be more effective than focusing on extrinsic rewards (e.g., feeling a sugar rush after eating a cupcake), which are fleeting and therefore feed the habitual process through wanting more. In the context of mindfulness training, the freedom that results from disentangling oneself from the demands of old habits and cravings opens a doorway to direct one’s energies to more fruitful pursuits, including simply savoring life’s moments (eating and otherwise). A mindfulness practice is itself reinforcing and may directly align with values and goals around healthy eating, with rewards that encourage further practice and development of insight which sustains long-term improvements in both mindfulness and healthy lifestyle choices.

As with any paradigm shift, critical questions remain. Namely, does the process of moving from extrinsic to intrinsic reward through mindfulness lead to long-term changes, independent of other lifestyle interventions (such as physical activity instruction or nutrition education)? At what point is it optimal to pair mindfulness training with an additional intervention and for whom? We would predict that mindfulness training in itself may afford reductions in reward-related eating, with consequent improvements in overall eating behavior. We also predict that it may be augmented when paired with nutritional strategies, yet that the timing of the pairing would be critical; bringing too many modalities together at once may overwhelm individuals rather than support them. In light of the considerable racial, ethnic, gender, and socioeconomic disparities across the range of food- and weight-related issues (e.g., Marques et al., 2011; Diggins et al., 2015; Krueger and Reither, 2015; Calzo et al., 2017), a critical next step is understanding how to disseminate mindfulness training to individuals from diverse backgrounds. Also, are mobile or web-based programs effective means of program implementation, or does the addition of in-person support (e.g., weekly facilitator led drop-in support groups) increase effectiveness? Future studies should seek to answer these questions in order to continue forward progress in the field of mindfulness and its effects on reward-related eating.

## Author Contributions

JB and AM contributed conception and design of the manuscript. JB wrote the first draft of the manuscript. AR, AB, GE, RvL, and AM wrote sections of the manuscript. All authors contributed to manuscript revision, read and approved the submitted version.

## Conflict of Interest Statement

JB is the founder of and owns stock in Claritas MindSciences, the company that developed the mindful eating app (Eat Right Now). He is also the research lead there. He has not received any payments or funding from the company for any work related this manuscript. The remaining authors declare that the research was conducted in the absence of any commercial or financial relationships that could be construed as a potential conflict of interest.
